# Impact of surgical case load on recurrence rates in pilonidal sinus disease: a cross-study data synthesis

**DOI:** 10.1007/s00384-025-04883-0

**Published:** 2025-05-23

**Authors:** Dietrich Doll, Matthias Maak, Ida Kaad Faurschou, Theo Hackmann, Christina Oetzmann von Sochaczewski, Myriam Braun-Münker, Igors Iesalnieks, Susanne Haas, the Pilonidal Network for Expertise, Research and Development (PiloNERDs International), Dietrich Doll, Matthias Maak, Susanne Haas, Ida Farschou, Helene Hougaard, Marlene Julia Sørensen, Anne Vestbjerg Thyø, Theo Hackmann, Henrike Heitmann, Sofia Barbosa, Marius Dettmer, Christina Oetzmann von Sochaczewski

**Affiliations:** 1 Department of Procto-Surgery & Pilonidal Sinus, St. Marienhospital Vechta, Academic Teaching Hospital of the MHH Hannover, Vechta, Germany; 2 Department of Surgery, University Hospital Erlangen of the Friedrich-Alexander University Erlangen-Nuremberg, Krankenhausstraße 12, 91054 Erlangen, Germany; 3 Department of Surgery, University Hospital of Bonn, Venusberg-Campus Bonn, Bonn, Germany; 4 Department of Surgery, Randers Regional Hospital, Randers, Denmark; 5 Department of Food Technology, Fulda University of Applied Sciences, Leipziger Straße 123, 36037 Fulda, Germany; 6 Department of Surgery, Evangelisches Krankenhaus Kalk, Cologne, Germany

**Keywords:** Pilonidal sinus, Therapy, Long term recurrence rate, Incidence, Observation bias, Case load, Operations per year

## Abstract

**Purpose:**

A higher surgical caseload has been associated with better outcomes in various diseases. However, this relationship has not yet been studied for Pilonidal Sinus Disease (PSD). This study aimed to examine the impact of annual PSD surgery volumes on recurrence rates (RR).

**Methods:**

A comparative cross-study data synthesis was conducted, including 1074 studies with 130,599 patients, focusing on PSD recurrence rates. Studies were categorized based on annual case load (ACL) into three groups: fewer than 10 operations per year (group 1), 10–30 operations per year (group 2), and 30 or more operations per year (group 3). Kaplan–Meier analyses were performed to evaluate recurrence rates, with additional stratification by study design (randomized controlled trial (RCT) or non-RCT) and surgical treatment type.

**Results:**

In randomized controlled trials (RCTs) of primary open treatment, group 1 (< 10 surgeries/year) had a 5-year recurrence rate of 23.5%, while group 2 (10–30 surgeries/year) had a rate of 12.5% (*p* < 0.0001). No data was available for group 3. For primary midline closure (pmc), the 5-year recurrence rates were 14.8% for group 1, 26.3% for group 2, and 12.8% for group 3. Non-RCT studies generally showed decreasing recurrence rates with higher surgical caseloads for most treatment types. However, midline closure surgery showed no significant improvement in recurrence rates with increased caseloads.

**Conclusion:**

The study demonstrates that higher surgical caseloads significantly reduce PSD recurrence rates, with notable benefits observed for centers treating 30 or more patients annually. Specialization in high-volume centers may lead to improved outcomes for PSD, particularly in flap surgeries. However, midline closure surgery continues to show persistently high recurrence rates, regardless of caseload.

**Supplementary Information:**

The online version contains supplementary material available at 10.1007/s00384-025-04883-0.

## Introduction

Pilonidal sinus disease (PSD) is a common but often overlooked surgical condition, primarily affecting adolescents [1, 2]. Patients frequently experience social stigma and uncertainty in seeking care, while PSD itself lacks a dedicated medical specialty. Management is dispersed across general, colorectal, plastic, and dermatologic surgeons, with no clear ownership, leading to inconsistencies in treatment approaches.

Anal and perianal surgical conditions are often low on medical priority lists, leaving pilonidal abscesses or fistulae to be handled by junior surgeons. While teaching opportunities exist, they are not common. Specialized clinics for teenagers with recurrent PSD provide more focused care, [3], compared to hospitals where surgeries are distributed among various practitioners.

Paradoxically, despite the significant patient population, the number of cases handled per physician varies greatly. In 2018, Nora Peters analyzed PSD treatment in German hospitals, revealing annual caseloads ranging from 1 to 160 patients per hospital, with a median of 38 operations per year—less than one operation per week [4].

Senior consultants (25%), junior consultants (24%), and registrars (27%) predominantly managed PSD cases during daytime hours, with department heads involved in 17% of cases. Notably, 24% of surgeries occurred at night, 10% of which were performed by department heads [5].

PSD surgeries are thus distributed across hospitals and performed by various departments or as outpatient procedures. While many physicians feel capable of managing PSD, training for advanced procedures remains limited and is often perceived as inadequate [6]. Regular training, with less than one operation per week, may be just sufficient [7], but the efficacy of such training remains unknown. Training younger doctors might necessitate further redistribution of surgical cases. Surgical approaches range from primary open and midline closures to rotation and advancement flaps (e.g., Limberg, Karydakis) and minimally invasive techniques like pit-picking, marsupialization, and laser ablation. Hospitals using multiple techniques often perform only a few cases per year per method, contributing to recurrence rates between 18 and 30% per year [5].

Although PSD surgery is technically not a rocket scince, its consequences can be significant, ranging from prolonged hospitalization and recovery to impaired sexual function [8, 9], frequent dressing changes, and the need for repeat surgeries [10]. Asymmetric closures and flap procedures consistently show the lowest recurrence rates [11–13], but mastering them requires experience. Some flap techniques, such as Karydakis, may require as few as 24 procedures to achieve proficiency [7, 14], raising concerns about whether surgeons receive adequate training across all methods, like rhomboid procedures, such as Limberg [15] and Dufourmentel [16].

Recognizing the interplay between decision-making and surgical skill, this study aimed to determine whether annual caseloads per study correlate with recurrence rates in PSD as reported in global literature. The null hypothesis (H₀) posited no correlation between annual caseloads and recurrence rates.

## Methods

**Fig. 1:**
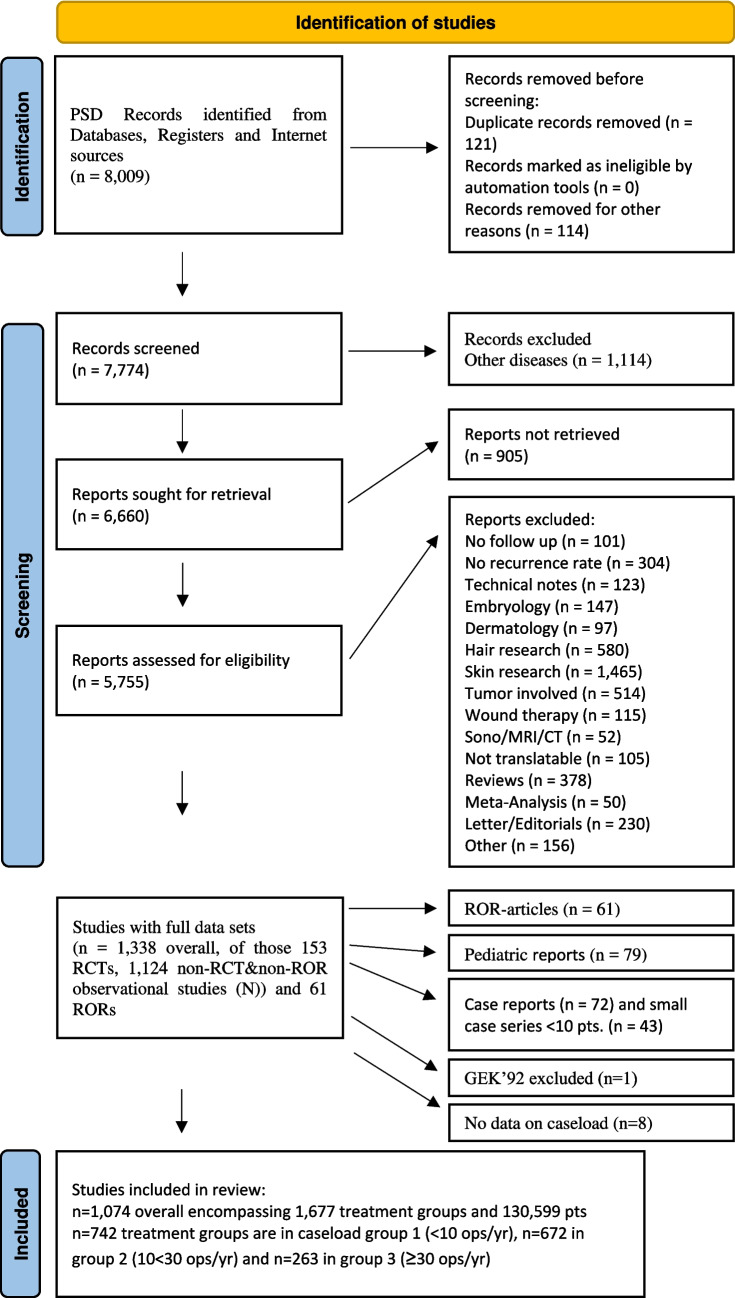
PRISMA flow chart of study harvest and qualification steps [21]. GEK’92 = George E. Karydakis study 1992

This study was conducted as part of a larger research project [17] and registered in the PROSPERO study register under the title, “The efficacy of the commonest surgical procedures in pilonidal sinus patients–a meta-analysis comparing the recurrence rates over time using long-term follow-up data” (PROSPERO 2016 CRD42016051588). The data retrieval process followed the Stauffer methodology [13], and the results are presented in the Preferred Reporting Items for Systematic Reviews and Meta-Analyses (PRISMA) flow diagram (Fig. 1).

### Search strategy and study selection criteria

A preliminary investigation that began in 1986 involved searching the German Bundeswehr Medical Archives (WEHRMEDSTATINST Bw Remagen) under the guidance of the archivists [18]. Using pinpoint search techniques, it was discovered that some non-PSD diseases had been incorrectly classified as PSD. Conversely, many PSD cases were misclassified under other conditions. By applying broader search terms, such as “pilonid*” and “dermoid*” combined with “cyst,” previously unrecognized PSD cases were identified and added to the dataset. This approach avoided reliance on ICD or OPS codes and significantly expanded the PSD population, though it required sifting through thousands of irrelevant findings. Ultimately, more than 5% of previously mislabeled reports were incorporated into the database. This methodology, akin to using a fine-mesh fishing net, was adopted to ensure thoroughness in building the PSD world literature database for subsequent research.

A systematic search of the global literature was conducted using the NCBI Medical Subject Heading (MeSH) term “pilonid*” and the combination “dermoid” AND “cyst.” This search covered all available databases, including MEDLINE, PubMed, PubMed Central, Scopus, Ovid, EMBASE, and the Cochrane Central Register of Controlled Trials (CENTRAL). Additional searches using these terms were performed on platforms like Google, Google Scholar, and ResearchGate. References cited in national and international guidelines, such as the S3 guidelines from the Association of the Scientific Medical Societies in Germany for PSD treatment, were also examined. Furthermore, bibliographies of all retrieved documents were reviewed.

The collected materials included a wide range of study types, such as randomized and non-randomized trials, prospective and retrospective studies, and observational studies (e.g., cohort studies, case–control studies, cross-sectional studies, and case reports). These documents spanned nearly two centuries, covering the period from 1833 to 2023.

Three authors (TH, HH, DD) meticulously reviewed the retrieved documents to ensure they met the inclusion criteria. These criteria required information on definitive treatment, recurrence rates, gender composition, and follow-up duration. Publications in English, French, German, Italian, and Spanish were included, as well as those in other languages if an English abstract provided details on treatment, recurrence, and follow-up time. When translations were needed, authors were contacted via email or ResearchGate, and, alternatively, two different translation software tools were used to translate relevant text passages.

Exclusion criteria included PSD cases occurring outside the sacral region, involvement of neoplastic conditions, and duplicate publications of the same data by the same author. Studies that lacked any part of the minimal dataset—comprising definitive treatment, recurrence, and follow-up information—were also excluded. While previous meta-analyses and review articles were not included in the study, their reference lists were carefully reviewed to identify additional evidence. Unpublished data presented within these reviews were also considered for inclusion.

### Data collection, extraction, and quality assessment

All identified studies underwent a comprehensive analysis and were meticulously documented. The extracted data were transcribed into a Microsoft Excel spreadsheet (Version 2016, Microsoft Corp., Redmond, WA, USA) and verified to ensure accuracy. Each distinct therapeutic strategy reported within a study was recorded on a separate line, with columns detailing citation information, the number of patients, therapeutic procedures, reported follow-up times, study characteristics, and recurrence data.

Due to the variability in statistical measures used to report follow-up times across studies, mean and median values were treated as equivalent, given that PSD predominantly affects young adults. When studies provided minimum follow-up times, these values were incorporated as reported.

Individual studies were systematically reviewed for methodological consistency and accuracy of reported results to minimize potential bias during data synthesis. A subgroup analysis of prospective randomized controlled trials (RCTs) was conducted to ensure alignment with the broader dataset. Recurrence rates from each study were linked to their corresponding follow-up times, whether reported as mean, median, the midpoint of a range, or minimum values.

To enable uniform comparisons across studies, individual patients were statistically simulated. Each study participant was represented as a data sample, capturing their recurrence status, follow-up time, and therapeutic procedure. For instance, if a study included 500 patients and reported a 20% recurrence rate for a specific therapeutic procedure, 100 samples were designated as having recurrent disease, while the remaining 400 were considered recurrence-free.

Certain variables, such as gender ratios, were excluded from the analysis since most studies reported these data in aggregate form. When an article discussed multiple therapeutic strategies, data for each treatment strategy were analyzed separately.

As pediatric pilonidal sinus disease exhibits distinct recurrence rate kinetics, only cohorts with a mean age of 18 years or older were included in this analysis [19]. Additional studies employing return-on-recurrence (ROR) follow-up were excluded due to their susceptibility to significant observational bias. This exclusion applied to PSD studies with ROR follow-up as well. Details of this selection process are outlined in the PRISMA schedule (Fig. 1).

As recommended by an expert group of statisticians, case reports and case series involving fewer than 10 patients were excluded from the analysis. A prior meta-regression analysis, conducted on a cohort of 89,583 patients, examined both established recurrence rate (RR) modifiers (such as choice of treatment, follow-up duration, age, and Methylene Blue use) and potentially influential variables (including year of therapy, country of treatment, and annual caseload). This analysis confirmed the impact and relative importance of the established factors.

Other potential influences, such as family history and recurrence status (primary versus recurrent disease), were inconsistently reported in the global literature and too limited to allow meaningful analysis. Similarly, cardiovascular comorbidities were rare, as PSD primarily affects patients aged 15 to 25 years. Previous co-interventions were also infrequently documented and, as a result, were not investigated.

For a detailed discussion of potential biases in worldwide database analyses, please refer to the following publication [17].

### Grouping of therapeutic procedures and statistical analyses

Therapeutic procedures were analyzed both collectively (“overall across study design”) and in subcategories based on specific surgical treatment methods.

Is it the total number of cases reported by the study per year of study duration?

The annual caseload calculated by dividing the number of cases for each surgical method by the duration of years of the study. These annual caseloads were then grouped into three categories: < 10 operations per year, 10– < 30 operations per year, and ≥ 30 operations per year. This grouping resulted in three cohorts comprising 24,165, 49,052, and 57,382 patients, respectively.

The decision to define the first group as < 10 operations per year was supported by literature indicating this level represents the early stages of a surgeon’s learning curve [20], as calculated from Australian data [7]. This group is also below the success rate threshold of one of the flap procedures [7]. The threshold of ≥ 30 operations per year was based on Wysocki’s findings, which demonstrated that proficiency in flap procedures could be achieved after performing 30 cases in a short period. Additionally, the annual caseload was further sub-grouped by surgical method.

To ensure data reliability, it was assumed that no hospital could publish data on successive patients treated with the same method more than once a year. After reviewing 100 cases of authors publishing twice annually, where different treatment groups were consistently reported, this assumption was validated. Double publications were excluded in accordance with previously outlined criteria.

### Grouping of therapeutic procedures, gender cohorts, and statistical analyses

Therapeutic procedures were analyzed for RCT and nonRCT and further subcategorized into therapeutic categories. Variants of therapeutic procedures were consolidated under their originating procedures, as outlined in Table 1).

**Table 1:** Log rank *p*-values für RCTs only, comparing the influence of annual case load on recurrence rate for different therapies (Figs. 6, 7, 8, 9, and 10)

Caseload p.a	Overall	Primary open	Midline closure	Bascom/Karyd	Limberg/Duf
< 10 vs. 10 < 30	*p* = 0.039	***p* < 0.0001**	*p* = 0.14	***p* = 0.0051**	***p* = 0.04825**
< 10 vs. ≥ 30	*p* = 0.8	***p* = 0.00078**	*p* = 0.14	***p* < 0.0001**	***p* = 0.00013**
10 < 30 vs. ≥ 30	*p* = 0.039	*p* = 0.05827	*p* = 0.14	***p* = 0.0051**	***p* = 0.04825**

significant values are given in bold

Statistical analysis and visualization were performed using the R statistical software package (version 4.3.2) within the RStudio framework (version 2023.6.1.524). Statistical significance was defined as *p* < 0.05, and all tests were two-tailed. Kaplan–Meier survival analysis was employed to assess recurrence-free outcomes over time, along with pointwise 95% confidence intervals (CI), using the R package *survival* (version 3.5–7). These analyses were conducted for both RCT and non-RCT cohorts, with results presented as the percentage of recurrence-free outcomes and corresponding 95% CIs. Graphical representations were created using the *survminer* package (version 0.4.9), and a two-tailed log-rank test was applied.

For two-group comparisons, Student’s *t*-test was used. The horizontal axes of the Kaplan–Meier plots indicated the number of patients within specific follow-up intervals, measured in months. Where data for specific intervals were unavailable, linear interpolation was applied to estimate recurrence-free outcomes based on the two nearest observed follow-up times. The analysis timeframe was truncated at 10 years postoperatively.

To provide a comprehensive assessment, data from both RCTs and non-RCTs were evaluated. Due to the incremental nature of Kaplan–Meier curves, minor discrepancies between plotted and tabulated values may occur.

## Results

**Fig. 2:**
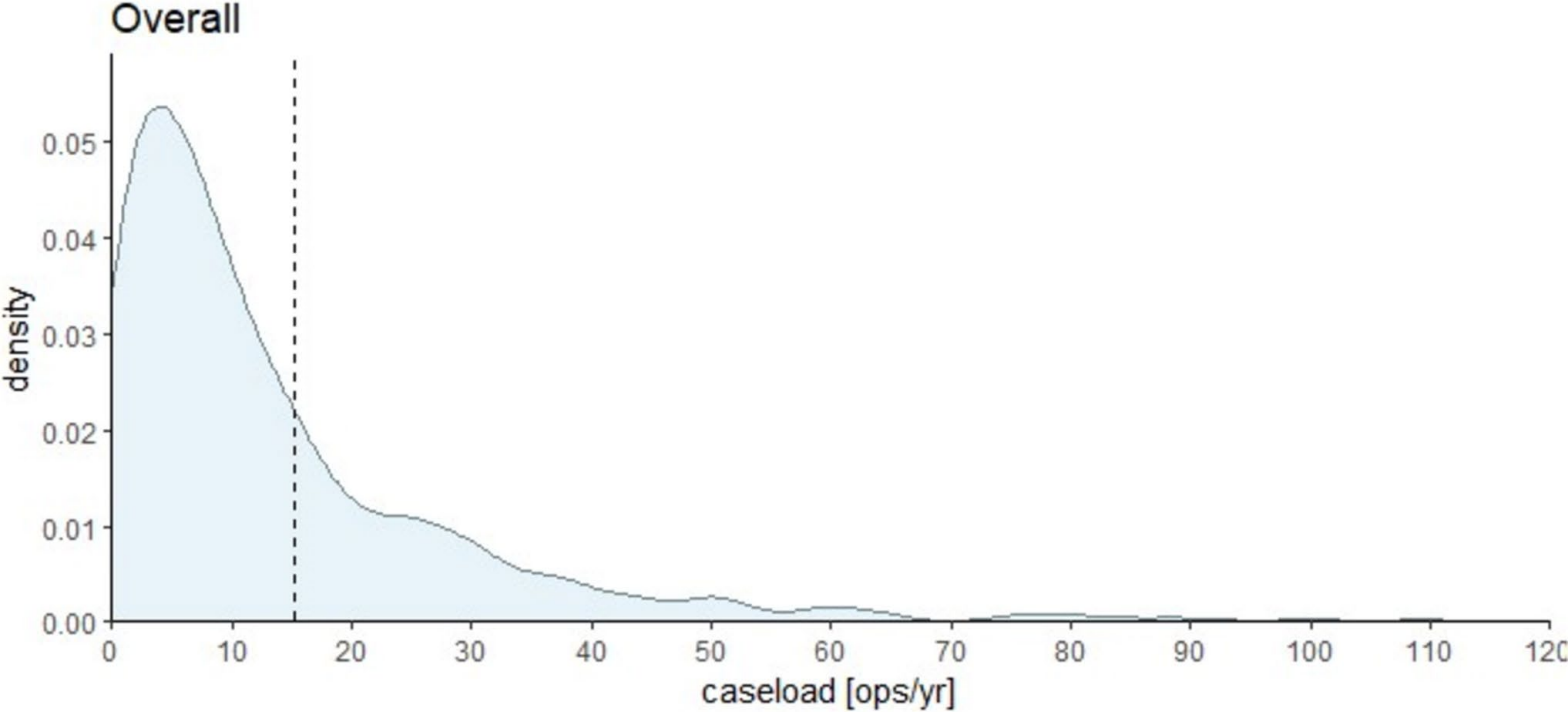
Histogram of annual case load per method; all methods together *n* = 130,599 pts (mean 16 ops p.a., median 9 ops p.a.)

In total, our analysis included 1074 studies involving 130,599 patients, spanning the years from 1833 to 2023. From these studies, we analyzed the caseload distribution, which is shown in the histogram (Fig. 2).

**Fig. 3:**
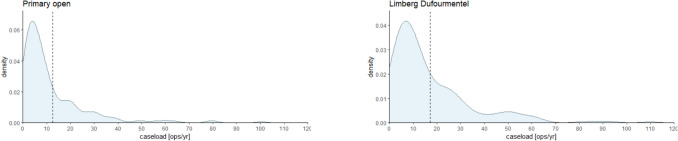

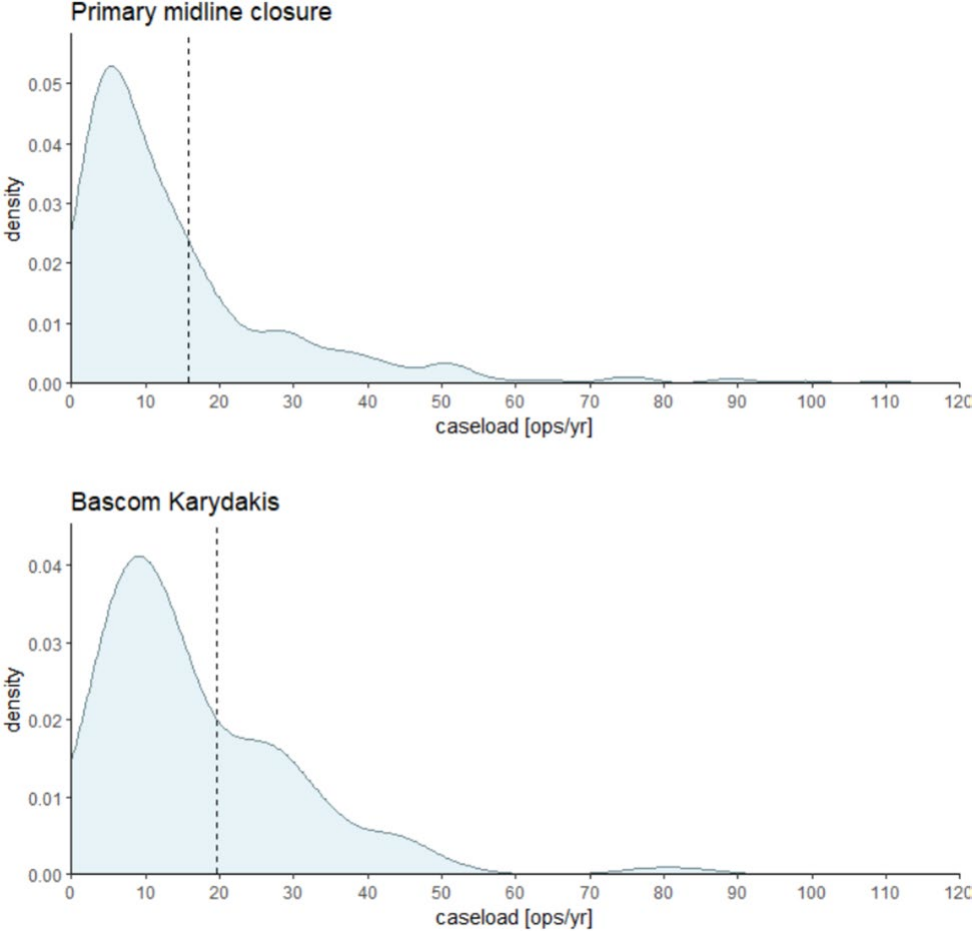
**a**–**d** Histogram of annual case load for each method of the most used surgical methods. Please note that the *Y*-axes use different scaling

The observed spectrum of caseload per methodology ranges from 1 to 882 patients published annually. The mean caseload per annum stands at 16 patients, with a median of 9 patients. Figure 3a–d illustrates the histogram delineating caseload distribution for the predominant surgical modalities in treating Pilonidal Sinus Disease (PSD).

As can be seen from the density plots for primary open wound treatment (Fig. 3a, above left), midline closure (Fig. 3b, above right), Limberg/Dufourmentel (Fig. 3c, below left), and Karydakis/Bascom (Fig. 3d, below right) and case load means/medians per year and method range between 16/9 (all), 15/7 (primary open), 17/9 (primary midline), 16/10 (Limberg/Dufourmentel), and 20/13 (Karydakis/Bascom) operations per year and study. Primary midline seems to be the most often used method in this cohort analyzed but applied and published in smaller cohorts (despite no longer being recommended) [22–24]. All curves in Fig. 3a–d peak between 5 and 14 operations representing the most common annual case load (ACL) per method worldwide.

**Fig. 4:**
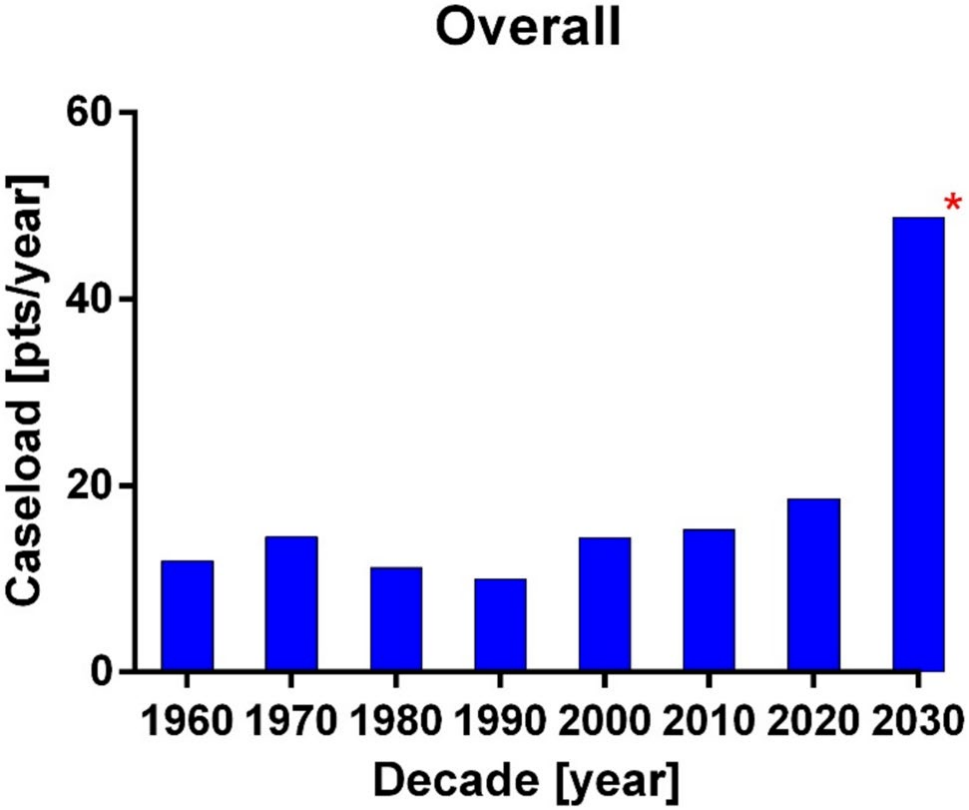
Annual case load histogram per decades; all methods. *The 2030 decade contains the years 2020 following and is incomplete at this time

When analyzing the trend of ACL reported over the last 30 years, an upward trend is apparent (Fig. 4). However, it is unclear whether this trend reflects an actual increase in the number of PSD cases being diagnosed and operated on each year, a result of better detection and treatment, or simply a greater volume of published PSD cases. Specialization of authors, attracting larger numbers of operations, may also be a contributing factor, which is a welcome development.

There is an overall upward trend of annual case load published for all methods combined until the 2020 decade (Fig. 4). It is important to note that the 2030 decade is not yet complete, so this column may be subject to future corrections as more data becomes available.

**Fig. 5:**
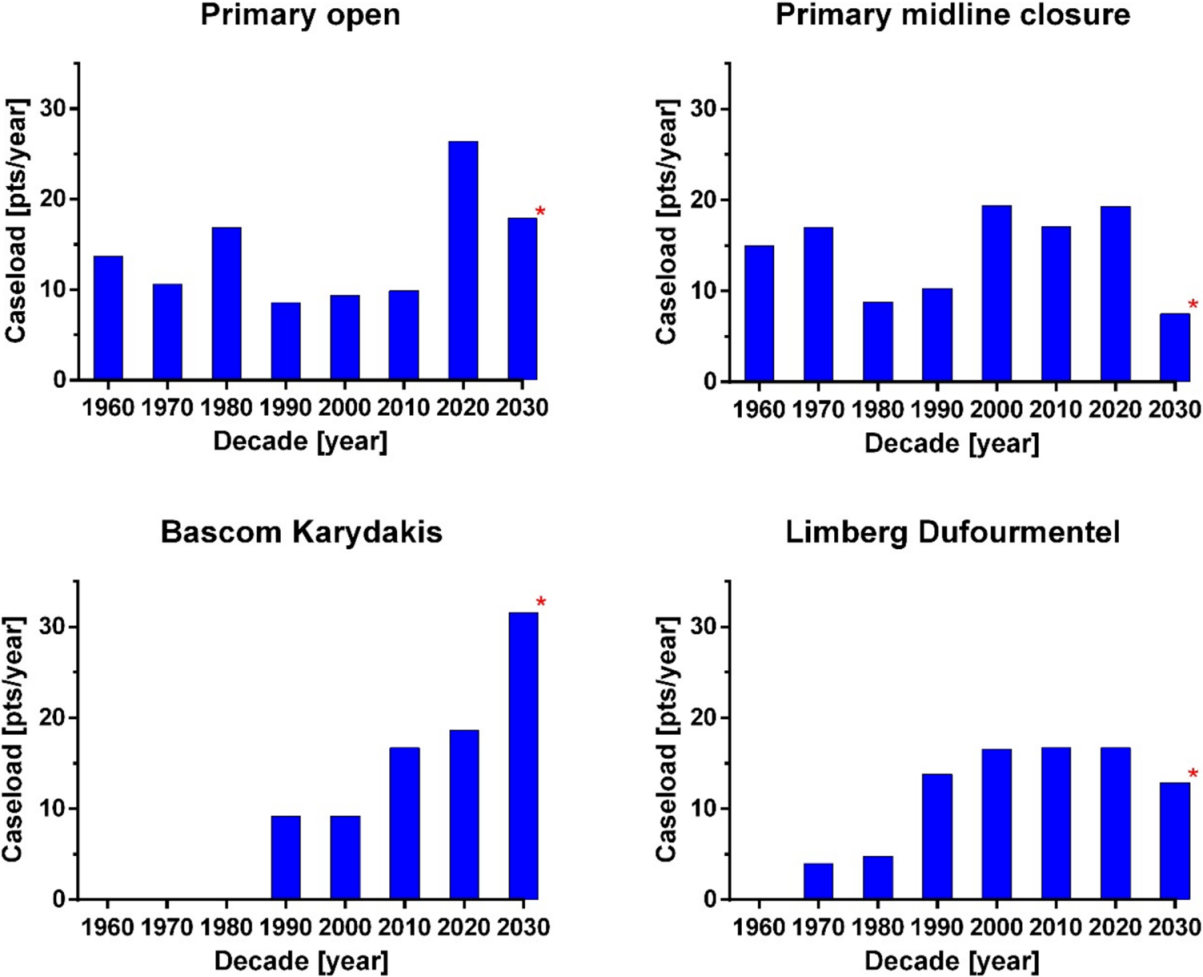
**a**–**d** Annual case load per decade, separate for the four most applied surgical methods. *The 2030 decade contains the years 2020 following and is incomplete at this time

Figure 5a–d shows the annual case load of these four methods. The annual case load (ACL) of flap methods Karydakis/Bascom (lower left) and Limberg/Dufourmentel (lower right) shows a substantial increase over the last four decades. Surprisingly, the primary open method (upper left) seems to be experiencing a revival, as larger ACLs per hospital are observed in the 2020 decade, which are more than twice as high compared to the previous three decades. Most surprisingly, primary midline therapy is still in use, with its application appearing to increase in the recent decades of 2000–2020, after a period of lower yearly case loads from 1960 to 1990. The highest ACL in the 2020 decade is observed in the primary open wound treatment group, while the incomplete 2030 data shows a trend toward Karydakis/Bascom flaps.

As outlined in the methods section, annual case load (ACL) was grouped into < 10 operations, 10–30 operations, and ≥ 30 operations per year to create equally sized groups, using cut-offs supported by the literature (*n* = 24,165 patients, *n* = 49,052 patients, and *n* = 57,382 patients, respectively) when analyzing all methods of both RCT and non-RCTs together. Since recurrence rates (RR) may differ between RCTs and non-RCTs [17], as noted in the literature, these are analyzed separately as follows.

## RCT studies

**Fig. 6:**
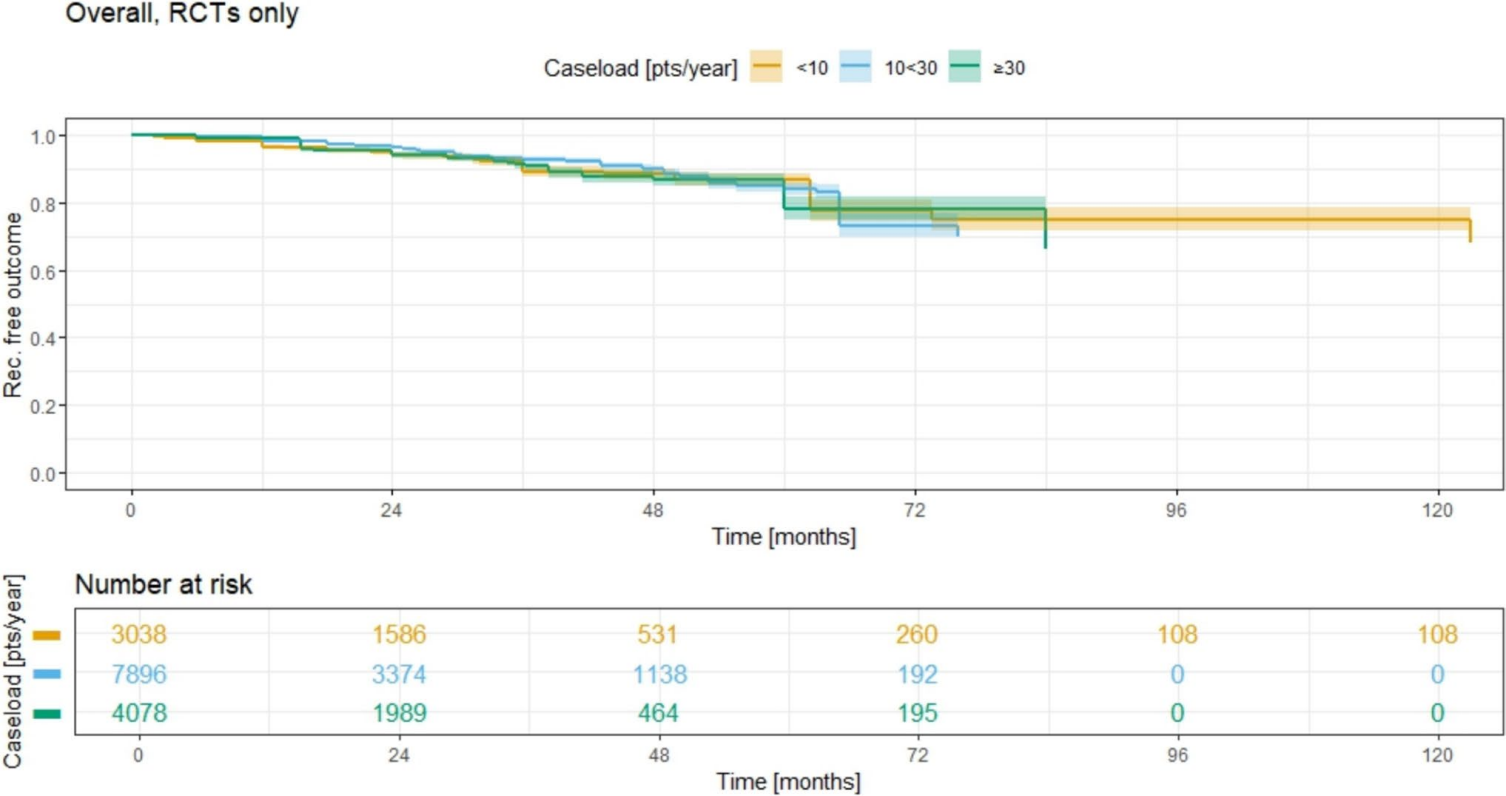
Recurrence free outcome for all methods combined and associated recurrence rate for different annual case load (ACL), RCTs only

In RCTs only analysis for all treatments (Fig. 6), the 5-year recurrence rates (RR) are 13.6% (95% CI 11.5–15.5) for ACL < 10 (group 1, yellow), 16.1% (95% CI 14.1–18.0) for ACL 10–30 (group 2, blue), and 21.8% (95% CI 18.5–25.0) for ACL ≥ 30 (group 3, green). The 10-year RR is only available for group 1 and is 25.1% (95% CI 21.5–28.5).

**Fig. 7:**
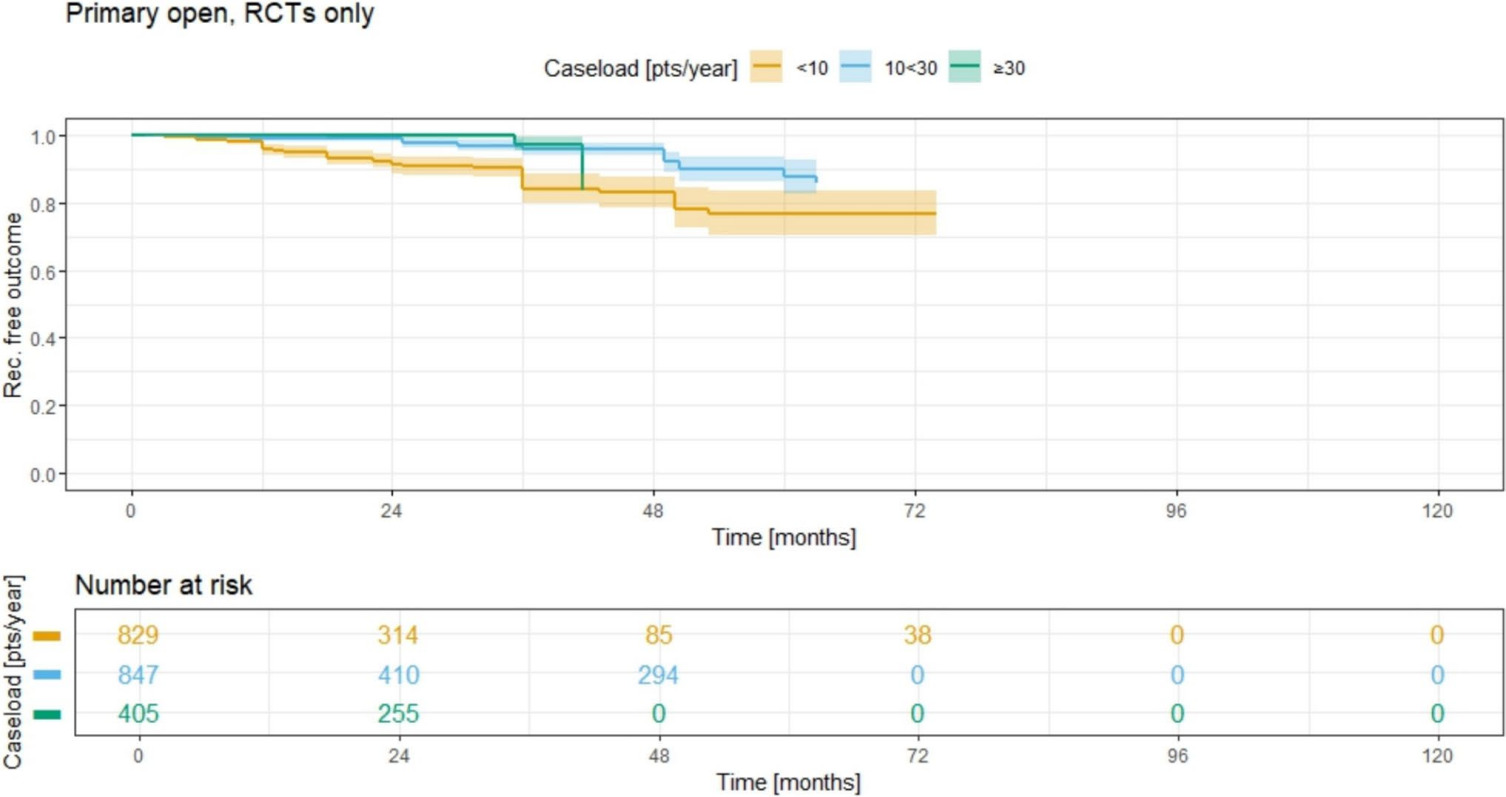
Recurrence free outcome for primary open method and associated recurrence rate for different ACLs; RCTs only

As shown in Fig. 7, the procedure-specific analysis for the primary open treatment group reveals a 5-year recurrence rate of 23.5% (95% CI 16.5–29.8) for group 1 and 12.5% (95% CI 7.6–17.7) for group 2. No data was available at 5 years of follow-up for group 3, and there was no data for any group at 10 years of follow-up.

**Fig. 8:**
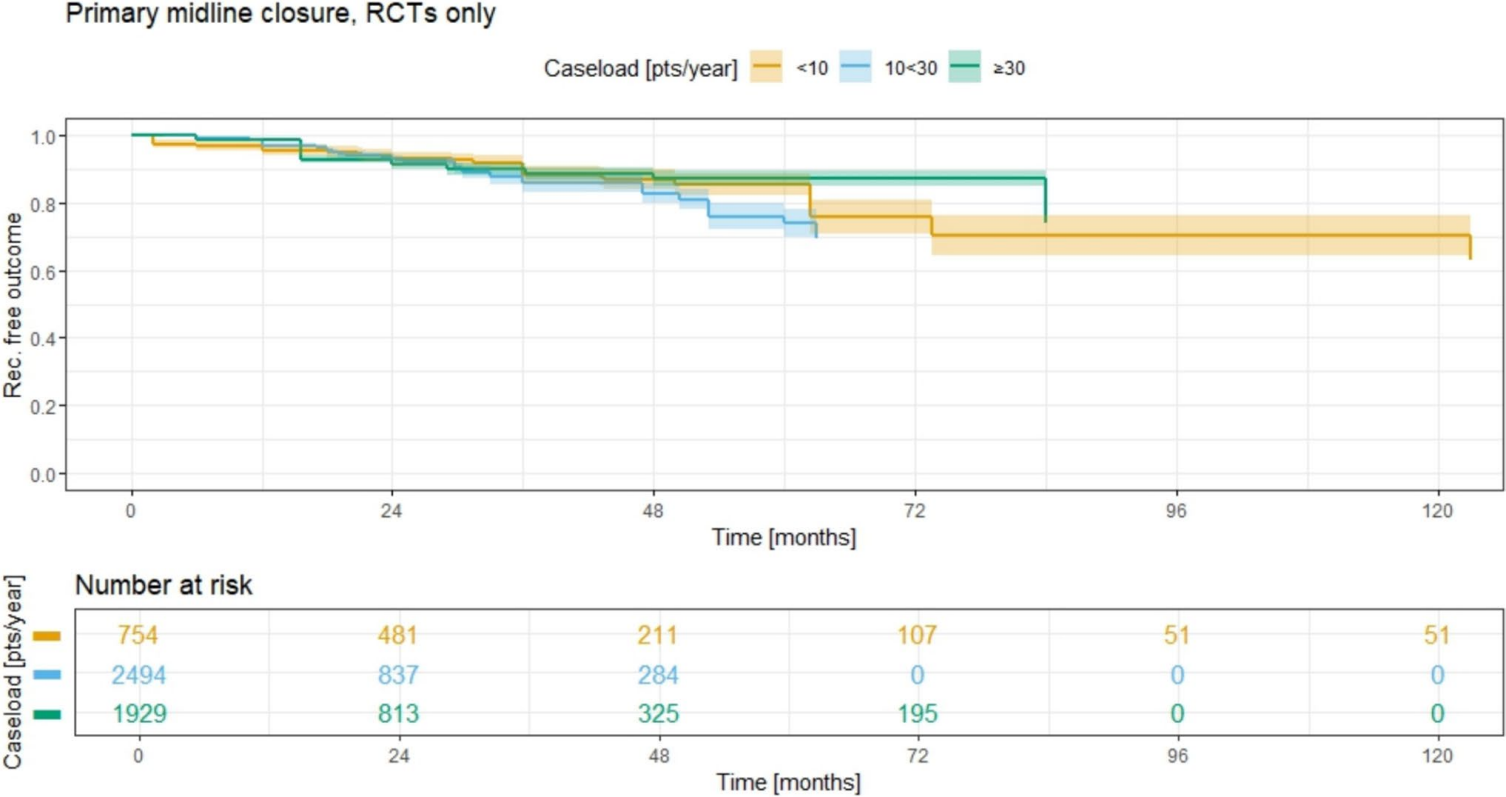
Recurrence free outcome for primary midline closure and associated recurrence rate for different ACLs; RCTs only

In the RCT analysis for primary midline closure (Fig. 8), the 5-year recurrence rates for groups 1–3 are 14.8% (95% CI 11.3–18.1), 26.3% (95% CI 22.0–30.4), and 12.8% (95% CI 10.6–15.0), respectively. The 10-year recurrence rate of 29.9% (95% CI 23.7–35.7) is only available for group 1.

**Fig. 9:**
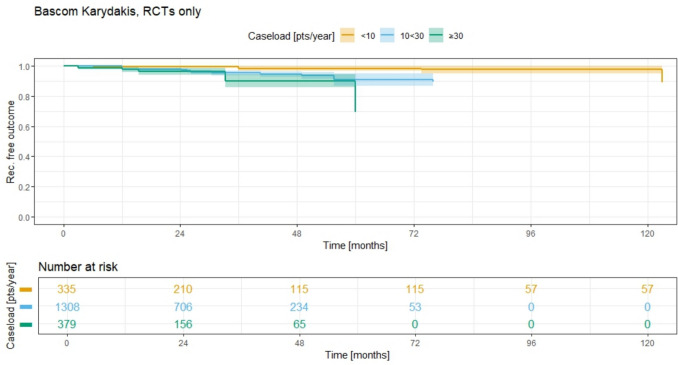
Recurrence free outcome for Bascom/Karydakis advancement flap and associated recurrence rate for different ACLs; RCTs only

Analysis of Bascom Karydakis treatment in RCT studies only (Fig. 9) reveals 5-year recurrence rates of 1.8% (95% CI 0.0–3.5%) for group 1, 9.1% (95% CI 4.9–13.1%) for group 2, and 30.7% (95% CI 20.2–39.9%) for group 3. The 10-year recurrence rate for group 1 is 2.6% (95% CI 0.2–5.0%), while no data are available for groups 2 and 3 at that time. Given the small study size for group 1, with limited data beyond 72 months, and the higher recurrence-free outcome in this group compared to groups 2 and 3, these results should be interpreted with caution.

**Fig. 10:**
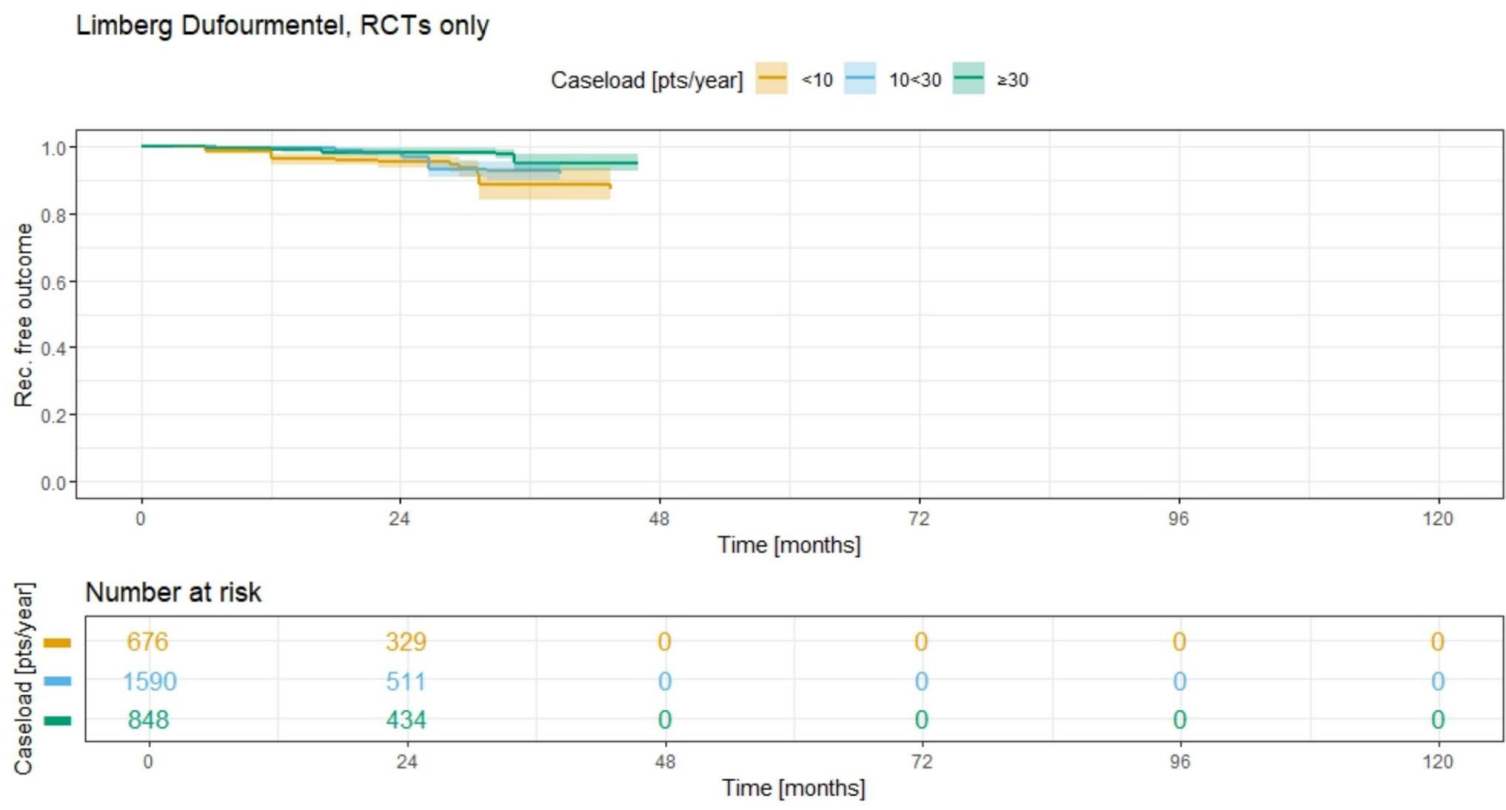
Recurrence free outcome for Limberg/Dufourmentel rhomboid rotation flap and associated recurrence rate for different ACLs; RCTs only

In RCT analysis of Limberg/Dufourmentel (Fig. 10), only short term results (less than 5 years) are available. As shown in Fig. 10, the 3-year recurrence rates (RR) are 11.4% (95% CI 6.5–16.0%) for group 1, 7.5% (95% CI 4.9–10.1%) for group 2, and 5.0% (95% CI 2.6–7.4%) for group 3 at 36 months. All three curves differ significantly, with group 3 showing the lowest recurrence rate, group 2 displaying an intermediate recurrence rate, and the group with the lowest annual case load (group 1) showing the highest recurrence rate among the three Limberg/Dufourmentel groups at that time. The results of log-rank testing for the RCT studies and their respective groups are displayed in Table 1.

## NonRCT studies

**Fig. 11:**
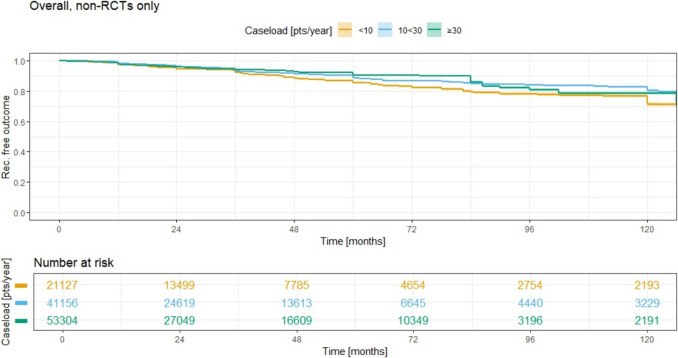
Annual case load per method and consecutive recurrence rate; all methods together in non-RCT analysis

Using Kaplan–Meier analysis on the larger non-RCT cohort with all therapy groups combined, the recurrence-free outcome was plotted against time since surgery (on the *x*-axis, in months) for the three annual case load (ACL) groups, as shown in Fig. 11. The dark green group (ACL ≥ 30) demonstrates the largest recurrence-free outcome, indicating the lowest recurrence rate over time since surgery.

The Kaplan–Meier analysis of the larger non-RCT cohort reveals distinct differences in recurrence rates across the three ACL groups, as shown in Fig. 11. The lowest line on the graph corresponds to group 1 (ACL < 10), which drops significantly below the other two groups, indicating that the recurrence rate is highest in the lowest ACL group. The 5-year and 10-year recurrence rates for ACL < 10 were 14.6% (95% CI 13.9–15.3) and 28.8% (95% CI 27.6–30.0), respectively. In contrast, the recurrence rates for the ACL 10 < 30 group were 11.4% (95% CI 11.0–11.9) at 5 years and 19.8% (95% CI 19.0–20.7) at 10 years. For the ACL ≥ 30 group, the 5-year recurrence rate was 9.7% (95% CI 9.3–10.1), and the 10-year rate was 21.6% (95% CI 20.7–22.6). All three curves differed significantly from each other (*p* < 0.0001; log rank test, Table 2.

**Table 2:** Log rank test *p*-values for nonRCT Kaplan–Meier analysis of case load vs recurrence curves from Figs. 11, 12, 13, 14, and 15

Caseload p.a	Overall	Primary open	Midline closure	Bascom/Karyd	Limberg/Duf
< 10 vs. 10 < 30	***p* < 0.0001**	***p* < 0.0001**	*p* = 0.13	*p* = 0.9	***p* = 0.032**
< 10 vs. ≥ 30	***p* < 0.0001**	***p* < 0.0001**	*p* = 0.85	*p* = 0.9	*p* = 0.525
10 < 30 vs. ≥ 30	***p* < 0.0001**	***p* < 0.0001**	*p* = 0.85	*p* = 0.9	***p* < 0.0001**

significant values are given in bold

Further analysis of different therapy subgroups within the non-RCT cohort shows that for the primary open group (Fig. 7): the ACL ≥ 30 group exhibited the best recurrence-free outcome (i.e., the lowest recurrence rate). For this group, the 5-year recurrence rate was as low as 3.1% (95% CI 2.5–3.8%). The ACL 10 < 30 group had a recurrence rate that was intermediate between the largest and smallest ACL groups, with a 5-year recurrence rate of 8.3% (95% CI 7.3–9.3) and a 10-year recurrence rate of 13.3% (95% CI 11.9–14.7). In contrast, the ACL < 10 group had the highest recurrence rate, with a 5-year rate of 12.0% (95% CI 10.4–13.5) and a 10-year rate of 24.0% (95% CI 21.2–26.7). Again, all three curves differed significantly (*p* < 0.0001; log rank test).

**Fig. 12:**
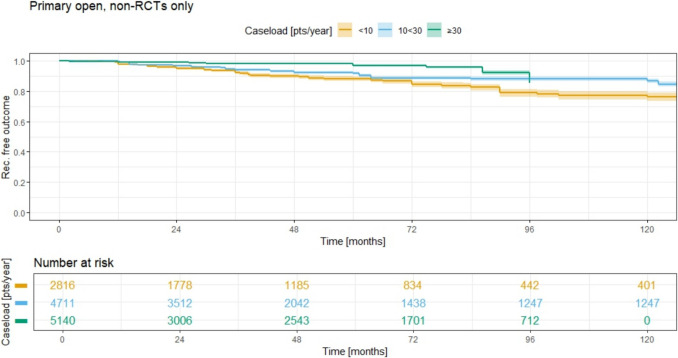
Annual case load for primary open method and consecutive recurrence rate; nonRCTs

These findings suggest that while higher caseloads (≥ 30 operations per year) seem statistically comparable in terms of long-term recurrence rates, the smallest group (ACL < 10) clearly stands apart, with higher recurrence rates both in the short- and long-term follow-up periods (Fig. 12).

**Fig. 13:**
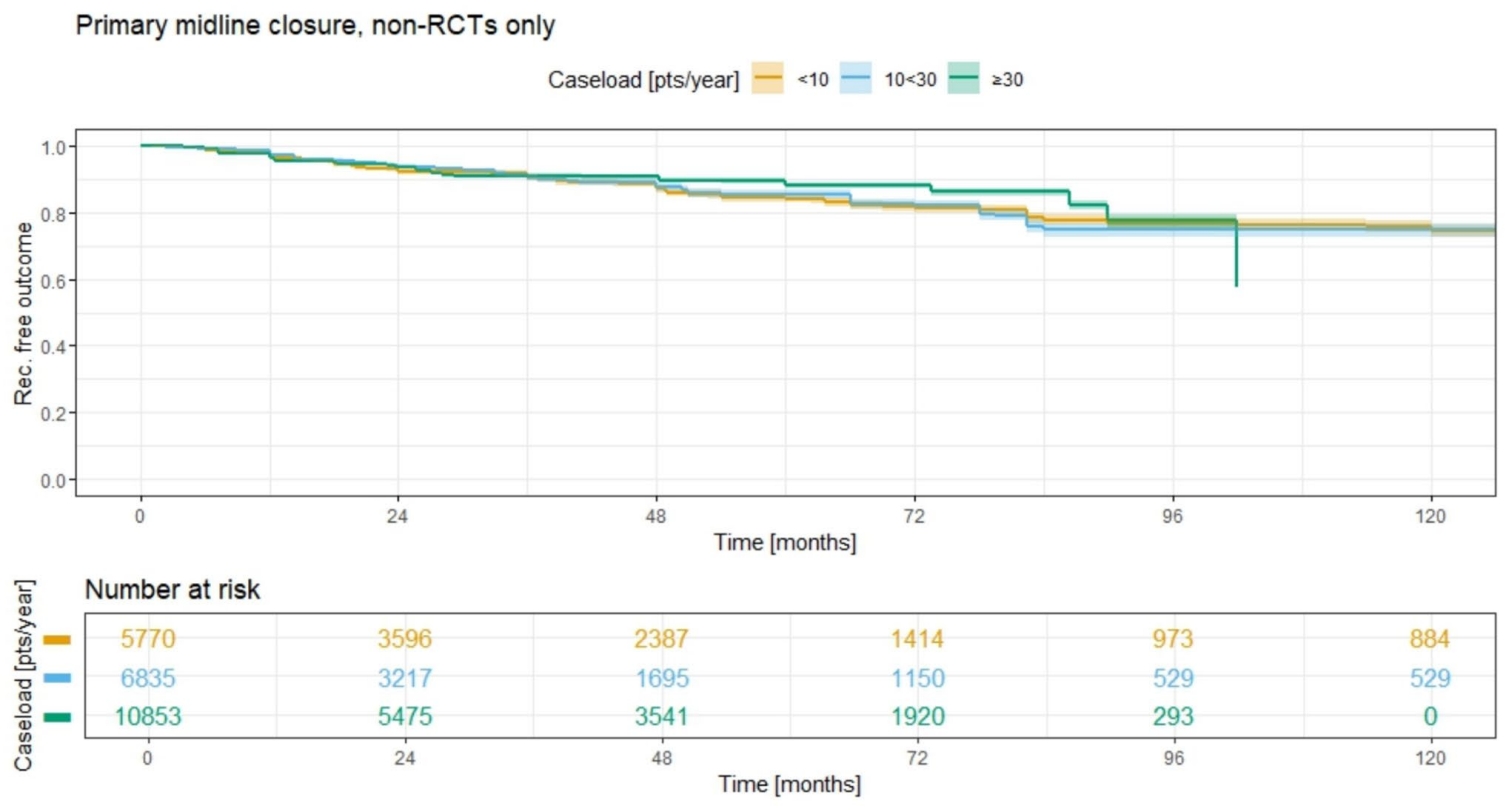
Annual case load for primary midline method and consecutive recurrence rate

The Kaplan–Meier analysis of recurrence rates for primary midline closure (pmc) (Fig. 13) reveals consistently high recurrence rates across all three ACL groups. The 5-year recurrence rates are 15.9% (95% CI 14.7–17.2) for group 1 (ACL < 10), 14.6% (95% CI 13.2–15.9) for group 2 (ACL 10 < 30), and 11.9% (95% CI 11.0–12.8) for group 3 (ACL ≥ 30). At 10 years, the recurrence rates remain similarly high, with group 1 showing a rate of 25.6% (95% CI 23.7–27.5) and group 2 showing 25.2% (95% CI 23.1–27.3). However, no data are available for group 3 at the 10-year follow-up. Notably, at 96 months, all three curves converge, indicating that, despite initial differences, the recurrence rates for the three ACL groups become similar over time.

**Fig. 14:**
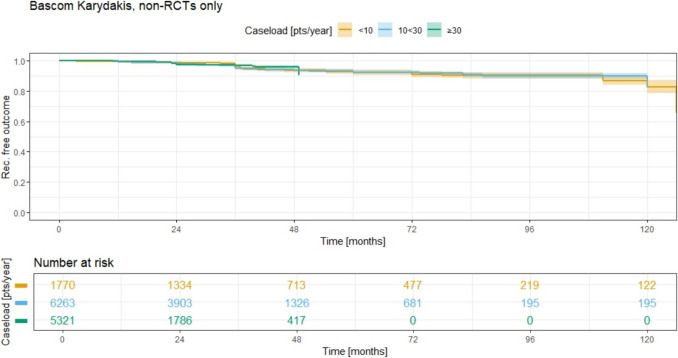
Annual case load for Bascom Karydakis method and consecutive recurrence rate

**Fig. 15:**
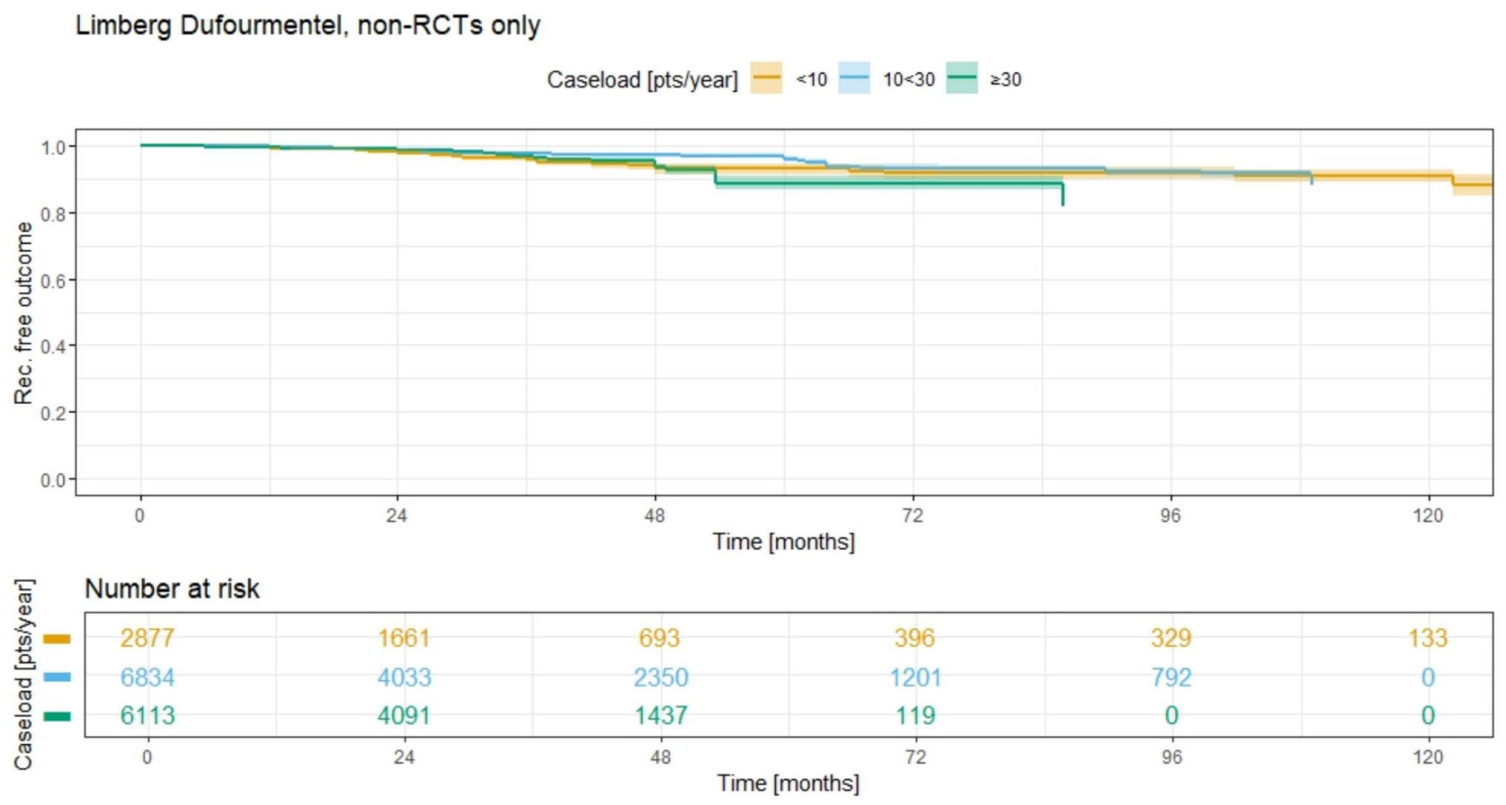
Annual case load for Limberg and Dufourmentel method and consecutive recurrence rate

In a similar pattern to the primary midline closure, the Kaplan–Meier curves for Bascom/Karydakis (Fig. 14) and Limberg/Dufourmentel (Fig. 15) therapies also remain closely aligned but show lower recurrence rates. For Bascom/Karydakis therapy, the 5-year recurrence rates are 8.0% (95% CI 6.2–9.6) for group 1 (ACL < 10), 7.7% (95% CI 6.6–8.8) for group 2 (ACL 10 < 30), and no data available for group 3 (ACL ≥ 30). The recurrence rates between these groups are statistically similar, with a *p*-value of 0.9 for all comparisons. In Limberg/Dufourmentel therapy, the 5-year recurrence rates are 7.2% (95% CI 5.7–8.6) for group 1 (ACL < 10), 4.2% (95% CI 3.5–4.9) for group 2 (ACL 10 < 30), and 11.5% (95% CI 9.4–13.5) for group 3 (ACL ≥ 30). The recurrence rates for these groups indicate some variation, with the group 2 (ACL 10 < 30) showing the lowest rate.

The *p*-values for the nonRCT comparison of the influence of annual case load (ACL) on recurrence rates across all therapies (“overall”), primary open therapy (po), Bascom/Karydakis, and Limberg/Dufourmentel therapies can be found in Table 2.

## Discussion

This is the most comprehensive cross-study data synthesis comparing the influence of annual case load (ACL) on the recurrence rate of pilonidal sinus disease (PSD) across multiple surgical techniques. Our findings underscore the importance of surgical volume in influencing recurrence rates of pilonidal sinus disease (PSD), particularly in low-volume hospitals. The analysis reveals that hospitals with an annual caseload (ACL) of fewer than 10 cases show significantly higher recurrence rates, and outcomes improve as the ACL increases, particularly in hospitals performing > 30 cases per year. However, while the differences observed were statistically significant, we acknowledge that the clinical significance of these findings, especially for high-volume centers, may not be as pronounced as initially expected. Notably, the results from randomized controlled trials (RCTs) show relatively smaller differences in recurrence rates between groups, suggesting that factors beyond surgical volume—such as technique refinement, surgeon experience, and patient-specific characteristics—also play a crucial role in outcomes. In particular, flap techniques like the Bascom/Karydakis and Limberg/Dufourmentel procedures showed reliably low recurrence rates, even when performed in centers with moderate volumes. This points to the fact that, once mastered, these techniques can maintain consistent outcomes, regardless of case volume, and highlights the role of surgical expertise in achieving favorable results.

However, primary midline closure—an outdated technique [11, 25–28]—consistently yields poor results, regardless of experience. This underscores the importance of both surgical expertise and technique selection in PSD management. Interestingly, while the 5-year and 10-year recurrence rates for the Bascom/Karydakis and Limberg/Dufourmentel techniques differ in RCT analysis, they remain consistent in the nonRCT analysis, with no apparent effect of annual case load (ACL) in our cohort of 29,178 patients. This pattern may be explained by a variety of factors, particularly the nature of flap or asymmetric techniques, which, despite being perceived as more complex due to potential complications [6], have seen an increase in use per hospital over time (Fig. 5c–d). This suggests that once surgeons master flap procedures, outcomes remain consistent, independent of volume. Flap techniques, though initially complex, are increasingly adopted and refined by dedicated specialists, reflecting broader acceptance and improved proficiency over time. This aligns with the rising number of records found in the databases when searching for these specific keywords, suggesting that the technique is becoming more mainstream.

Selective publication bias appears unlikely, as recurrence rates have remained stable over five decades despite rising PSD incidence [29]. If publication bias were a major influence, we would expect to see more exaggerated positive results. Instead, the stable recurrence rates, coupled with the increasing use of flap techniques, support the notion that these procedures may indeed be improving over time, independent of case load. Interestingly, prior surgical interventions did not clearly influence recurrence rates in this study. Methylene blue (MB), commonly used for intraoperative tract delineation, leading to larger excisions, longer wound healing [30], and contributing to lower recurrence rates [31], was evenly distributed across ACL groups, suggesting it is not a confounding factor.

Wysocki’s findings [7] support the notion that supervised training in flap surgery significantly improves outcomes. In contrast, midline closure continues to yield poor results in adults. Despite numerous studies highlighting its ineffectiveness, midline closure is still performed in about 10% of German hospitals [5], with similar practices observed in Austria, Switzerland, and the UK [32, 33]. In Denmark, the use of midline closure has been decreasing, partly due to national training initiatives promoting alternative methods such as pit picking and cleft lift surgery [34].

## Limitations

This study is limited by its retrospective design and heterogeneity among included studies. This approach incorporated both randomized controlled trials (RCTs) and non-RCT studies, intentionally excluding return-on-recurrence studies due to their potential observational bias [35].

Mean recurrence rates were analyzed at different time points, and Kaplan–Meier data were not consistently available. As a result, heterogeneity exists within the dataset, prompting us to adapt our statistical methods accordingly.

It is important to acknowledge that surgeons likely treat a larger number of patients than is reflected in their published figures, suggesting that their practical expertise may exceed the scope of the reported cohorts. Additionally, individual surgeon volumes could not be determined, as PSD surgeries are performed by surgeons of varying experience levels within hospitals, therefore it not possible to determine how the cases are distributed in a unit performing 30 cases per year.

Patient-related factors allegedly related to recurrence—such as smoking, diabetes, BMI, hair removal, and lifestyle—were not examined, though their random distribution likely minimizes bias. Furthermore, poor outcomes in primary open surgery may stem from inadequate technique, such as incomplete tract resection or improper wound management, or later embedding of hair into the long-time open wound causing future recurrences, adding to the effects of a low annual case load.

Our results suggest there is potential for improvement in the primary open technique, particularly in hospitals with lower PSD case loads, as indicated by our data. Several factors may contribute to suboptimal outcomes in primary open treatment, such as failure to apply methylene blue, improper resection of all tracts, or unnecessarily excising the wound too close to the anus. Acute abscess-forming disease, for instance, benefits from two-stage surgery [36] and shows a lower recurrence rate when performed during the daytime [37]. A common error in primary open treatment is exposing the sacral fascia, a practice that has been discouraged since the early twentieth century [30, 38, 39]. This exposure allows skin and wound bacteria to access the fascia, a bradytrophic tissue with limited blood supply, which leads to infection and delayed healing. Prolonged inflammation in this tissue reduces its ability to combat infection and initiate the necessary processes for tissue repair and wound healing. Furthermore, partial closure or leaving the wound open can facilitate the entry of hair, potentially leading to recurrent disease.

Kaplan et al. [40] found that prolonged healing times increased recurrence risk, particularly in younger patients. Similarly, Demiryas and Donmez [41] linked wound infections and dehiscence to higher recurrence rates, raising the question of whether less experienced surgeons create larger wounds, increasing infection risks. Alternatively, other technical factors may contribute to the observed higher infection and recurrence rates in such cases. [42].

## Implications and conclusion

Our findings highlight the need for specialization and possibly centralization of PSD treatment, similar to Denmark’s restructuring of colorectal cancer care [43–45]. Establishing national and international standards for surgical volume and quality monitoring could improve outcomes [10, 30, 37–42].

This cross-study data synthesis of 130,599 patients confirms that annual case load significantly influences recurrence rates, particularly in low-volume hospitals. While high ACL improves outcomes, flap surgery remains effective even at moderate volumes when performed by trained specialists. The continued use of midline closure should be abandoned in favor of evidence-based techniques. Ultimately, surgical expertise—guided by experience and proper training—is the key determinant of PSD treatment success.

## Supplementary Information

Below is the link to the electronic supplementary material.

Supplementary file1 (DOCX 17 KB)

Supplementary file2 (PDF 248 KB)

Supplementary file3 (DOCX 31 KB)

## Data Availability

No datasets were generated or analysed during the current study.
