# Supplementary material for: Impact of surgical case load on recurrence rates in pilonidal sinus disease: a cross-study data synthesis
Source: Int J Colorectal Dis. 2025 May 23;40(1):126. doi: 10.1007/s00384-025-04883-0 (PMC12102125; doi:10.1007/s00384-025-04883-0)
Supplement: Supplementary file 1 — Supplementary file1 (DOCX 17 KB) [file 384_2025_4883_MOESM1_ESM.docx]

***Impact of Surgical Case Load on Recurrence Rates in Pilonidal Sinus Disease:***

***A Comprehensive Meta-Analysis***

**AMSTAR 2 checklist**

**Item 1: Did the research questions and inclusion criteria for the review include the components of PICO?**

Yes, as far as applicable.

**Item 2: Did the report of the review contain an explicit statement that the review methods were established prior to conduct of the review and did the report justify any significant deviations from the protocol?**

Yes.

This study was conducted as part of a larger research project[18] and registered in the PROSPERO study register under the title, “The efficacy of the commonest surgical procedures in pilonidal sinus patients – a meta-analysis comparing the recurrence rates over time using long-term follow-up data” (PROSPERO 2016 CRD42016051588

(page 5)

**Item 3: Did the review authors explain their selection of the study designs for inclusion in the review?**

Yes

(page 6)

**Item 4: Did the review authors use a comprehensive literature search strategy?**

Yes.

This search covered all available databases, including MEDLINE, PubMed, PubMed Central, Scopus, Ovid, EMBASE, and the Cochrane Central Register of Controlled Trials (CENTRAL).

Publications in English, French, German, Italian, and Spanish were included, as well as those in other languages if an English abstract provided details on treatment, recurrence, and follow-up time. When translations were needed, authors were contacted via email or ResearchGate, and, alternatively, two different translation software tools were used to translate relevant text passages.

(page 6)

**Item 5: Did the review authors perform study selection in duplicate?**

Yes, to be precise, in triplicate. Three authors (TH, HH, DD) meticulously reviewed the retrieved documents to ensure they met the inclusion criteria.

(page 6)

**Item 6: Did the review authors perform data extraction in duplicate?**

Yes, of course. We are a small team.

**Item 7: Did the review authors provide a list of excluded studies and justify the exclusions?**

Yes, although not in detail. The Prisma flow chart on page 10 shows the reasons for exclusion. Of initial 8009 identified records just 1075 with full data sets where considered eligible for the study.

**Item 8: Did the review authors describe the included studies in adequate detail?**

Yes. As detailed as possible.

**Item 9: Did the review authors use a satisfactory technique for assessing the risk of bias**

Yes

Individual studies were systematically reviewed for methodological consistency and accuracy of reported results to minimize potential bias during data synthesis.

(page 7)

**Item 10: Did the review authors report on the sources of funding for the studies included in the review?**

Yes

No funding or grants from any other funding agencies in the public, commercial, or not-for-profit sectors were received.

(page 2)

**Item 11: If meta-analysis was justified did the review authors use appropriate methods for statistical combination of results? (Only complete this item if meta-analysis of other data synthesis techniques were reported)**

Yes.

**Item 12: If meta-analysis was performed did the review authors assess the potential impact of RoB in individual studies on the results of the meta-analysis or other evidence synthesis?**

Yes; see following question.

**Item 13: Did the review authors account for RoB in individual studies when interpreting/ discussing the results of the review?**

*Ex ante*, during the first steps of study analysis, any study with missing or unclear core data (large RoB) was excluded. Thus RoB was assessed initially, but not discussed at a later stage due to the sheer amount of studies analysed.

**Item 14: Did the review authors provide a satisfactory explanation for, and discussion of, any heterogeneity observed in the results of the review?**

Yes

**Item 15: If they performed quantitative synthesis did the review authors carry out an adequate investigation of publication bias (small study bias) and discuss its likely impact on the results of the review?**

We performed a world-all-in analysis, with studies exceeding 7,000 patients. In fact, at the smaller end, following statistical advice, we excluded case reports and case series smaller 10 patients. These studies were analysed separately and were shown to underperform in terms of recurrence rate accuracy (published <https://doi.org/10.1007/s00053-024-00825-6> ).

**Item 16: Did the review authors report any potential sources of conflict of interest, including any funding they received for conducting the review?**

Yes.

All authors declare that they have no conflicts of interest. There are no relevant or minor financial relationships between relatives or next of kin and external companies.

(page 2)
